# The Sulfur Monoxide–Water Complex

**DOI:** 10.1021/jacs.6c08436

**Published:** 2026-07-08

**Authors:** Guohai Deng, Stephen M. Goodlett, Caio M. Porto, Artur Mardyukov, Peter R. Schreiner

**Affiliations:** Institute of Organic Chemistry, 9175 Justus Liebig University, Heinrich-Buff-Ring 17, 35392 Giessen, Germany

## Abstract

The hitherto experimentally unreported chalcogen-bonded sulfur monoxide–water complex (OS···OH_2_), a key intermediate in atmospheric but even more so in interstellar SO chemistry, was generated through 254 nm photolysis of *in situ* generated metastable sulfoxylic acid (HOSOH) in argon matrices at 3.5 K. The acid was prepared in the gas phase through flash vacuum pyrolysis of *tert*-butylsulfinic acid (*t*-BuS­(O)­OH) at 600 °C. The characterization of HOSOH and the OS···OH_2_ complex was accomplished by means of matrix isolation IR and UV/vis spectroscopy and ^2^H-isotope labeling experiments. Focal point computations indicate the triplet sulfur monoxide–water complex with chalcogen bonding (OS···OH_2_) is 8.1 kcal mol^–1^ higher in energy than singlet sulfoxylic acid (HOSOH) and more stable than the triplet hydrogen bonding complex (SO···H_2_O) by 0.4 kcal mol^–1^ but only after inclusion of the zero-point vibrational energy correction.

## Graphical abstract

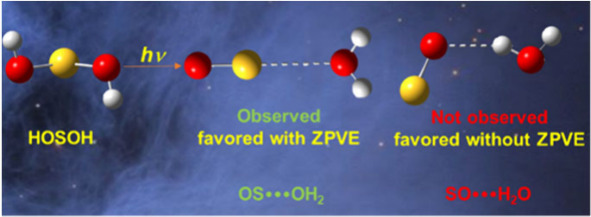

Sulfur monoxide (SO), isovalent to molecular oxygen, is the first intermediate arising from the oxidation of elemental sulfur. It forms in the atmosphere by direct oxidation^1^ or through reversible UV photolysis (190–220 nm) of SO_2_ (SO + O), a reaction believed to be responsible for the sulfur isotope mass independent fractionation in Earth’s atmosphere.^2–7^ The pathway for the generation of SO from photodecomposition of H_2_SO_4_ via SO_3_ and SO_2_ has been well established.^4,8^ In the Venusian atmosphere, highly reactive SO serves as a key intermediate that can form from UV-photolysis of SO_2_ or from volcanic eruptions (just as on Earth).^9,10^ Chemically, SO can undergo fast reactions with dienes and *N*-heterocyclic carbenes to produce unsaturated cyclic sulfoxides and sulfines, respectively.^11–14^ Due to its triplet ground state but in contrast to triplet O_2_, SO easily dimerizes in the gas phase and is further oxidized rapidly by molecular oxygen.^15,16^ In addition to dimerization and oxidation, its reactions with HCl, HF, and HO^•^ produce other sulfur-bearing species such as HOSCl (SO···HCl),^17^ HOSF (SO···HF),^17^ and HOSO^•^ (SO···HO)^18^ that have been investigated by matrix isolation spectroscopy and computational means.

Although the formation of H_2_O and SO in the gas phase has been proposed through the interaction of H_2_S with O_2_ or the dissociation of dihydroxysulfane (sulfoxylic acid),^19,20^ the interaction of SO with water remains only theoretically explored.^21,22^ According to the most recent UCCSD­(T)/aug-cc-pV­(Q+d)­Z,^22^ two minimum energy complexes should exist between SO and water. While one complex is hydrogen bonded (SO···H_2_O), the other displays a chalcogen bond (OS···OH_2_). The hydrogen bonded complex has a dissociation energy (*D*_e_) of +2.7 kcal mol^–1^, while the chalcogen bonded complex has a dissociation energy (*D*_e_) of +2.6 kcal mol^–1^. However, adding the zero-point vibrational energy (ZPVE) correction gives the experimentally more relevant *D*_0_ and the relative stabilities of the hydrogen vs chalcogen-bonded complexes change to *D*_0_ = 1.7 and 2.0 kcal mol^–1^, respectively. This is an intriguing theoretical prediction that has been rationalized based on the higher polarizability of sulfur vs oxygen, leading to more structural diversity for the heavier congener. As a consequence, the hydrogen-bonded O_2_···H_2_O complex only has a computed *D*_0_ of −0.7 kcal mol^–1^; chalcogen-bound complex is not even a minimum.^23^

As reactive water complexes, also including H_2_O···HNS/HSN,^24^ H_2_O···NH,^25^ H_2_O···NH_2_,^26^ H_2_O···HO,^27^ H_2_O···HOO,^28^ and H_2_O···SO_2_,^29^ are relevant to atmospheric and related processes as key intermediates en route to the reactive species’ water addition products, we reveal here the theoretically predicted but experimentally uncharacterized chalcogen-bonded sulfur monoxide–water complex (OS···OH_2_).

Sulfoxylic acid *C*_2_-**1** was prepared in the gas phase by flash vacuum pyrolysis (FVP) of *tert*-butylsulfinic acid (*t*-BuS­(O)­OH) (**2**) at 600 °C (Scheme 1). Specifically, **2** was evaporated at 26 °C and subjected to pyrolysis in a water-cooled quartz tube. The pyrolysis products were mixed with excess argon gas before being condensed onto a matrix CsI window at 3.5 K and serve as the molecular precursor to the OS···OH_2_ complex (*C*_2*v*_-**3**).

**Scheme 1:**
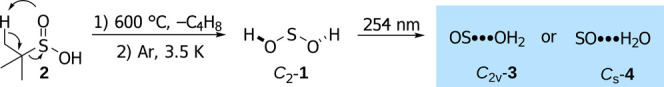
Sulfoxylic Scid HOSOH (*C*_2_-**1**) Generated from *tert*-Butylsulfinic Acid (**2**) through Pyrolysis and Trapping in an Argon Matrix and Subsequent Photoisomerization to the OS···OH_2_ Complex (*C*_2*v*_-**3**)­^a^

a Complex *C*_*s*_-**4** is not observed.

The IR spectrum of the pyrolysis products of *t*-BuS­(O)­OH isolated in an Ar-matrix is shown in Figure S1B. In addition to the strong bands of isobutene (b) and SO_2_ (d), as well as some weak bands of H_2_O (a), CO_2_ (c), and *t*-BuS­(O)­OH (**2**), a new product (*C*_2_-**1**) was detected and assigned (Table 1). Note that the band positions for HOSOH (3568–3554, 791, and 781 cm^–1^) have been reported in solid *para*-H_2_ matrix.^30^ The strong band at 777.0 cm^–1^ assigned to the characteristic SO stretching mode is blue-shifted compared to CH_3_SOH (766.4 cm^–1^)^31^ and NH_2_SOH (720.6 cm^–1^),^24^ due to the electron-withdrawing hydroxy substituent. The anharmonic stretching vibrations of the OH moiety in **1** is red-shifted compared to NH_2_SOH (3587.8 cm^–1^),^24^ CH_3_SOH (3588.3 cm^–1^),^31^ HCSOH (3583.4 cm^–1^),^32^ HSOH (3606.0 cm^–1^),^33^ and HONCO (3610.3 cm^–1^).^34^ The identification of *C*_2_-**1** and *C*_1_-^2^H-**1** is further supported by the agreement of the computed frequencies and ^2^H-isotopic shifts with the experimental values (Figure 1 and Table 1). For comparison, the computed structure and vibrational data of *C*_*s*_-**1** are given in Figure S2 and Table S1.

**Figure 1:**
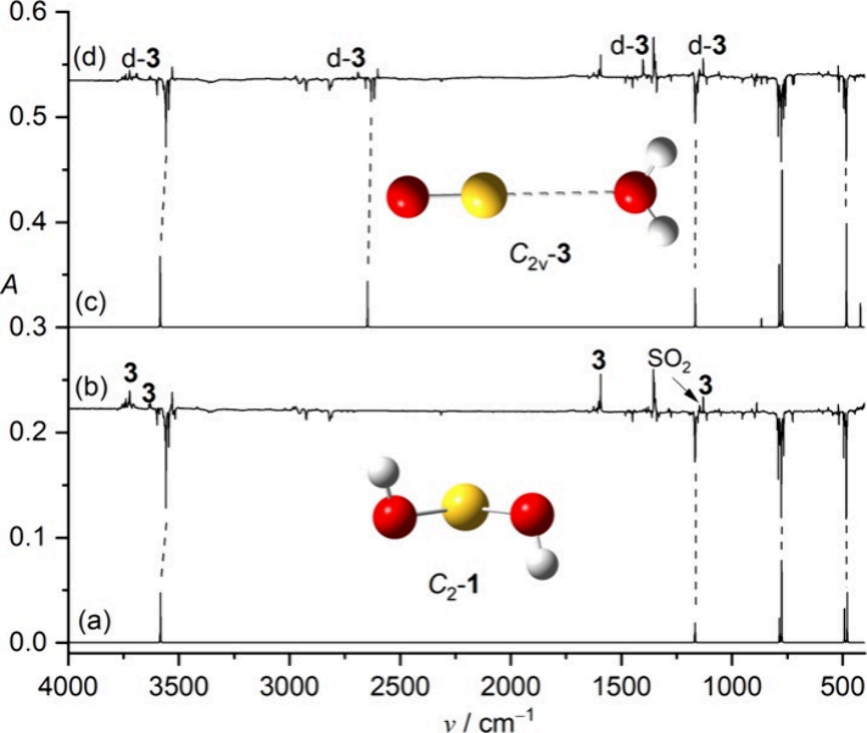
Infrared IR spectra showing the pyrolysis products of **2** with subsequent trapping in an argon matrix (3.5 K). (a) Computed IR spectrum of *C*_2_-**1** (CCSD­(T)/aug-cc-pV­(T+d)­Z corrected VPT2; see Supporting Information). (b) IR difference spectra showing *C*_2_-**1** after irradiation (λ = 254 nm). Downward *C*_2_-**1** bands disappear while upward *C*_2*v*_-**3** bands appear after 30 min irradiation. (c) Computed IR spectrum of *C*_1_-^2^H-**1**. (d) IR difference spectra of *C*_1_-^2^H-**1** after irradiation (λ = 254 nm). Downward *C*_1_-^2^H-**1** bands disappear and upward *C*_1_-^2^H-**3** bands appear after 30 min irradiation.

**Table 1:** Experimental and Computed IR Frequencies of *C*_2_-1 and *C*_1_-^2^H-1 (Singly Deuterated *C*_2_-1) Using CCSD­(T)/aug-cc-pV­(T+d)­Z Corrected VPT2 [Band Origins (cm^–1^) and Computed Intensities (km mol^–1^) in Parentheses]^a^

	*C*_2_-**1**		*C*_1_-^2^H-**1**		
sym.	theor.	Ar, 3.5 K	theor.	Ar, 3.5 K	assignment
*A*	3584.9 (18)	3560.7 vw	3583.8 (70)	3559.4 w	OH str.
*B*	3582.5 (123)	3557.9 s	2646.9 (44)	2630.8 w	O(H/D) str.
*B*	1168.5 (23)	1167.6 w	1165.8 (37)	1162.8 w	SOH in-plane bend
*A*	1165.9 (49)	1165.7 w	866.7 (9)	866.8 vw	SO(H/D) in-plane bend
*A*	785.3 (58)	790.1 w	786.0 (62)	789.5 w	SO str.
*B*	775.0 (196)	777.0 vs	773.0 (155)	775.6 vs	SO str.
*B*	491.0 (84)	494.8 s	481.9 (103)	485.9 s	SOH out-of-plane bend
*A*	478.6 (120)	482.7 s	418.8 (23)	^b^	SOH out-of-plane bend

a Assignments that change upon deuteration indicated with H/D. Band intensities: vs, very strong; s, strong; w, weak; and vw, very weak.

b Not observed.

Irradiation of *C*_2_-**1** in argon at 254 nm for 30 min results in decomposition and formation of new IR bands (Table 2) corresponding to a complex of SO with H_2_O (*C*_2*v*_-**3**). These bands are in agreement with the computed anharmonic IR frequencies (Table 2) and can therefore be assigned to *C*_2*v*_-**3**. The first three bands belong to the stretching and bending modes of H_2_O, which are shifted by −11.4, −108.1, and +4.9 cm^–1^ for the symmetric stretch, antisymmetric stretch, and bend respectively, compared to the absorption bands of free H_2_O in a solid Ar-matrix.^35^ The last band, attributed to the SO stretching mode in *C*_2*v*_-**3**, is red-shifted by −8.8 cm^–1^ with respect to the IR band of the triplet SO monomer at 1138.4 cm^–1^.^16^ This shift differs from that in other hydrogen-bonded complexes of SO such as SO···HO (+8.1 cm^–1^),^18^ SO···HCl (+4.0 cm^–1^),^17^ and SO···HF (+14.1 cm^–1^),^17^ in which SO acts as the hydrogen bond acceptor. However, it is similar to that in the SO_2_···H_2_O complex (−11.5 cm^–1^ and −2.2 cm^–1^).^29^ The computed SO vibration in the hydrogen bond complex SO···H_2_O is 1159.9 cm^–1^ (Table S2), which does not match the experimental value; hence, this complex is not present in our experiments. The formation of OS···OH_2_ is due to the matrix cage not allowing the photofragments SO and H_2_O to escape.^24,34^ The photochemistry of *C*_2_-**1** is similar to that of *C*_2*v*_-symmetric *s-trans*,*s-trans*-dihydroxycarbene HOCOH, which decomposes into a CO···H_2_O complex upon light irradiation.^36^

**Table 2:** Experimental and Computed IR Frequencies of *C*_2*v*_-**3** and *C*_*s*_-^2^H-**3** (Singly Deuterated *C*_2*v*_-3) Using CCSD­(T)/aug-cc-pV­(T+d)­Z Corrected VPT2 [Band Origins (cm^–1^) and Computed Intensities (km mol^–1^) in Parentheses]^a^

	*C*_2*v*_-**3**		*C*_*s*_-^2^H-**3**		
sym.	theor.	Ar, 3.5 K	theor.	Ar, 3.5 K	assignment
*B*_2_	3732.5 (108)	3732.1 s	3687.5 (57)	3693.2 w	OH str.
*A*_1_	3638.1 (6)	3630.0 vw	2706.4 (24)	2690.2 w	O(H/D) str.
*A*_1_	1599.1 (49)	1593.9 w	1406.5 (48)	1401.8 s	HO(H/D) bend
*A*_1_	1132.3 (68)	1129.6 w	1132.3 (68)	1129.6 vw	SO str.

a Assignments that change upon deuteration indicated with H/D. The first OH stretch corresponds to the antisymmetric stretch of water and the second OH stretch to the symmetric. Band intensities: s, strong; w, weak; and vw, very weak.

The UV–vis spectrum of *C*_2_-**1** was obtained in an Ar matrix (Figure 2). Bands at 193 and 223 nm were found for the *t*-BuS­(O)­OH precursor. Decomposition products were detected with absorption bands at 192 and 238 nm, as well as a broad band centered around 300 nm. The intensities of the 192, 238, and 300 nm bands decrease upon irradiation at 254 nm, giving rise to a new weak absorption band at 232 nm, assigned to *C*_2*v*_-**3** in agreement with EOM-CCSD computed SOMO-LUMO π → σ* transitions at 237 nm (Table S3).

**Figure 2:**
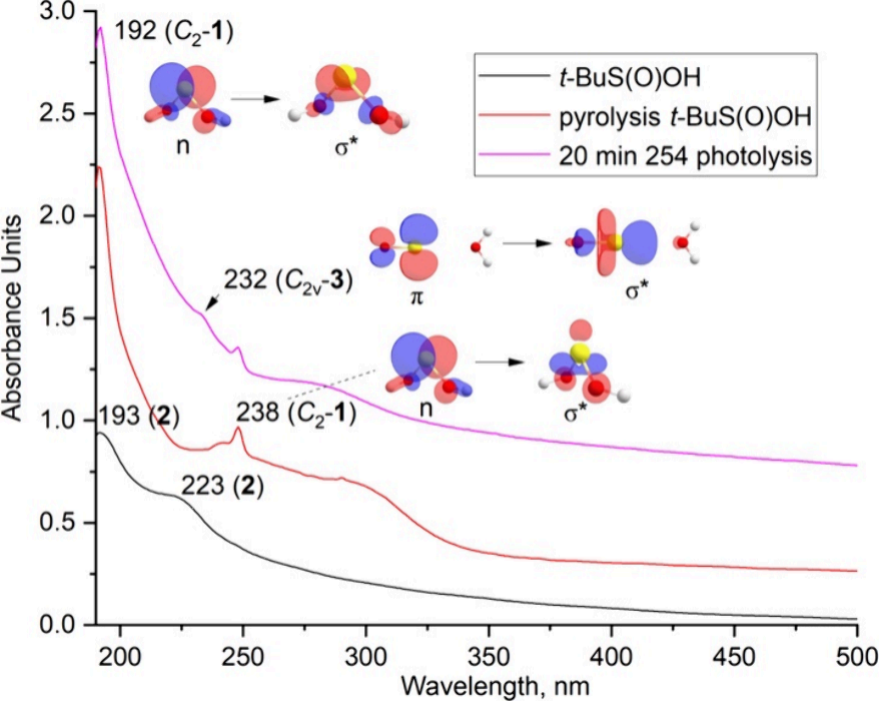
UV–vis spectra of matrix-isolated *t*-BuS­(O)­OH and the pyrolysis and subsequent photolysis products. Transition orbitals determined by EOM-CCSD/aug-cc-pV­(T+d)­Z.

CCSD­(T)/aug-cc-pV­(T+d)­Z optimized structures of **1** and the sulfur monoxide–water complexes *C*_2*v*_-**3** and *C*_*s*_-**4** are shown in Figures 3 and S2 and are consistent with previous theoretical reports.^20,22,37–40^ According to the computed energies using the focal point approach^41–43^ (Table S4), the most stable isomer *C*_2_-**1** has a singlet ground state with hydrogens *trans* to one another, while the *cis* isomer *C*_*s*_-**1** lies +1.2 kcal mol^–1^ above *C*_2_-**1**.

**Figure 3:**
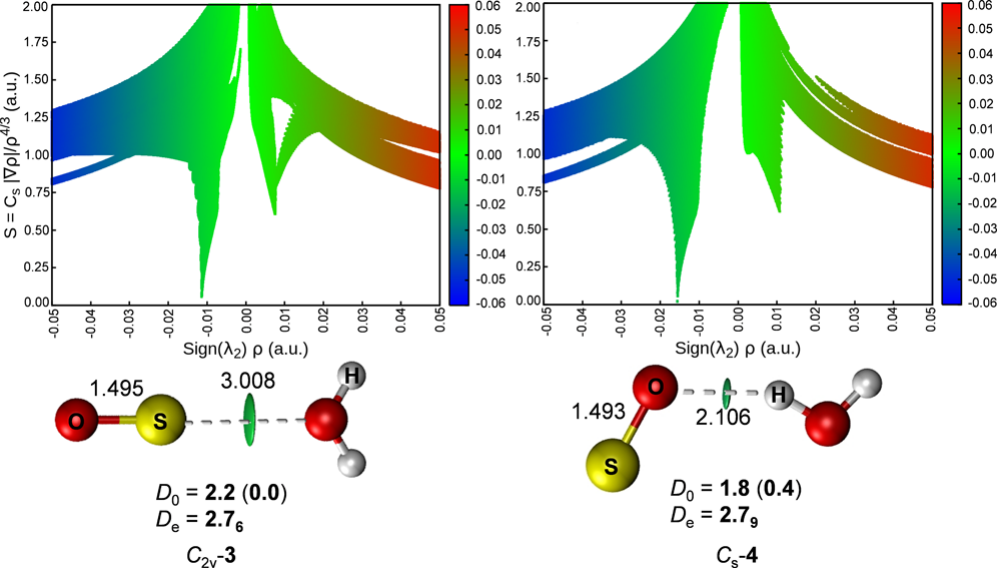
CCSD­(T)/aug-cc-pV­(T+d)­Z structures and focal point dissociation energies (kcal mol^–1^) (relative energies in parentheses) for the *C*_2*v*_-**3** and *C*_*s*_-**4** complexes. Reduced gradient versus the electron density (ρ) multiplied by the sign of the second Hessian eigenvalue (λ_2_) at B3LYP-D3­(BJ)/def2-TZVP shown for each species.

In agreement with Schaefer et al.,^22^ we located two triplet minima between SO and H_2_O, interacting via a chalcogen (OS···OH_2_, *C*_2*v*_-**3**) or hydrogen bond (SO···H_2_O, *C*_*s*_-**4**) (Scheme 1, Figure 3). In line with the experimental observation, focal point computations indicate *C*_2*v*_-**3** is −0.4 kcal mol^–1^ lower in energy than *C*_*s*_-**4**, with a dissociation energy (*D*_0_) of +2.2 kcal mol^–1^. When the ZPVE is not included, *C*_2*v*_-**3** is computed to be higher in energy than *C*_*s*_-**4** by 0.03 kcal mol^–1^, suggesting that the ZPVE drives the difference in the stability of the two species (*vide infra*).^22^ The anharmonic correction to the ZPVE contribution is likewise larger than might be expected (0.1 kcal mol^–1^) but does not change the relative energy ordering (Table S4). Local energy decomposition analysis^44,45^ (Figure S3) indicates *C*_*s*_-**4** has stronger electrostatic, exchange, and dispersion attractions than *C*_2*v*_-**3**. However, this is counterbalanced by a larger electronic preparation energy. These interactions are distance-dependent, so we can rationalize the interaction energies with a geometric argument. The computed chalcogen bond length for *C*_2*v*_-**3** is 3.008 Å (similar to O_2_S···OH_2_: 2.879 Å, MP2/6-311++G*),^29^ and the O···H bond distance for *C*_*s*_-**4** is 2.106 Å. Subtracting the Bondi radii (Rowland–Taylor for hydrogen)^46,47^ of the interacting atoms (H, 1.10; O, 1.52; S, 1.80 Å) gives a radial overlap of 0.312 and 0.514 Å for *C*_2*v*_-**3** and *C*_*s*_-**4**, respectively. The shorter effective interaction of *C*_*s*_-**4** helps explain the stronger LED contributions. Noncovalent interaction (NCI) analysis^48^ of *C*_2*v*_-**3** (Figure 3) features a stabilizing S···O interaction (λ_2_ = −0.0192; ρ = 0.0113), while *C*_*s*_-**4** resembles a hydrogen-bonded complex (λ_2_ = −0.0085; ρ = −0.0156). Natural bonding orbital analysis^49^ reveals the dominant orbital interactions are a water lone-pair to the SO antibond (n → σ*) donation for *C*_2*v*_-**3**, and SO oxygen lone pair to water OH antibond (n → σ*) donation for *C*_*s*_-**4**.

Note the delicate effect of including the ZPVE.^22^ Ignoring the ZPVE often results in a favorable cancellation of errors; however, the inclusion of the ZPVE is paramount to obtaining the correct energetic ordering of *C*_2*v*_-**3** and *C*_*s*_-**4**. Figure 4 investigates this sensitivity by comparing computed energy differences when using a “lower level” of theory for the geometry optimization and ZPVE computation. The overall trend is that a CCSD­(T) energy correction with the ZPVE included gives quantitative accuracy for most considered methods. Without the coupled cluster correction, MP2 is the only method to compute the wrong energy ordering even with the ZPVE, although many other methods show a similar magnitude of error in the opposite direction. Without the ZPVE, coupled cluster corrected energies give improper ordering for all considered methods.

**Figure 4:**
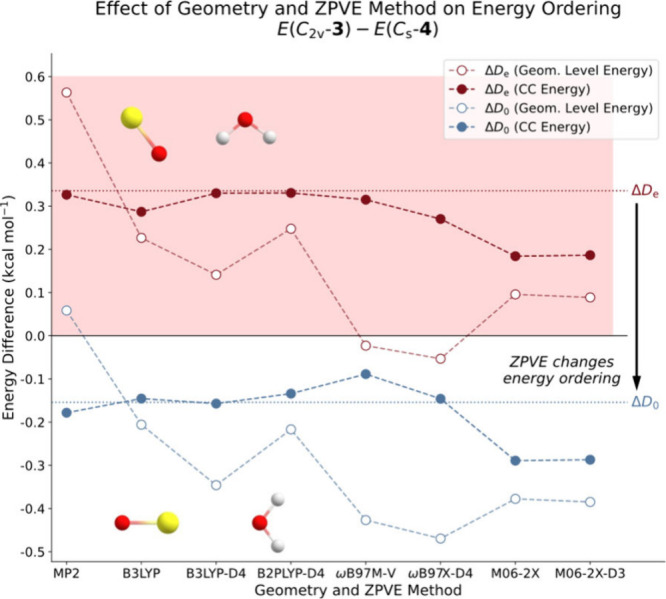
Relative energy difference between *C*_2*v*_-**3** and *C*_*s*_-**4** for several methods. Negative numbers indicate preference for experimentally observed *C*_2*v*_-**3**, and zero would indicate energetic equivalence. Horizontal lines indicate the CCSD­(T)/aug-cc-pV­(T+d)­Z values computed with (Δ*D*_0_, blue) and without (Δ*D*_e_, red) ZPVE corrections. The effect of including CCSD­(T) energy correction (filled) or not (unfilled) is also shown.

The results of Figure 4 motivate using B3LYP-D4/aug-cc-pV­(T+d)­Z to study other species on the reaction potential energy surfaces. The potential energy profile for the decomposition of *C*_2_-**1** was explored using CCSD­(T)/aug-cc-pVTZ//B3LYP-D4/aug-cc-pV­(T+d)­Z (Figure 5). Vertical excitation energies are also computed using NEVPT2­(6e,6o)^50,51^ /aug-cc-pV­(T+d)­Z. The lowest-energy pathway to *C*_2*v*_-**3** is via 1,3-hydrogen migration (**TS1**) with concerted S–O bond cleavage on the ground singlet surface. The formation of *C*_2*v*_-**3** could also proceed on the triplet manifold, after an intersystem crossing (ISC) from the excited singlet state, with transition states resembling **TS1**, or via **TS3** and **TS4**. Likely candidates for ISC from the excited singlet to triplet surface (where the energy of the two states become similar) are from the triplet minima of *C*_2_-**1** to **TS1**, or around the OS­(H)···OH structure. The absence of HOSO^•^ and HOS^•^ in the experimental infrared spectra is in accordance with the large bond dissociation energies for the O–H and S–O bonds of *C*_2_-**1**.

**Figure 5:**
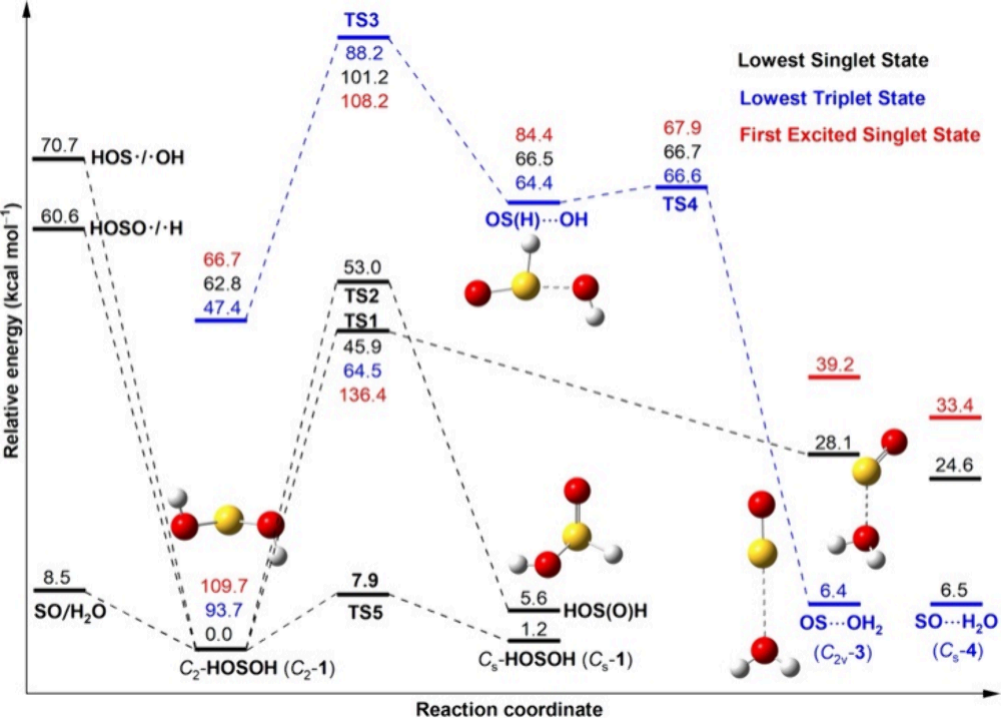
Computed potential energy profile (Δ*H*_0_) for the decomposition of *C*_2_-**1** with CCSD­(T)/aug-cc-pV­(T+d)­Z//B3LYP-D4/aug-cc-pV­(T+d)­Z. Black structures are optimized as singlets, and blue structures are optimized as triplets. NEVPT2­(6e,6o)/aug-cc-pV­(T+d)­Z is used to obtain excited state energies (lowest singlet, black; lowest triplet, blue; first excited singlet, red), and these are added to the CCSD­(T) energy to profile the excited state surfaces.

In conclusion, the theoretically predicted but experimentally elusive sulfur monoxide–water complex OS···OH_2_ (*C*_2*v*_-**3**) was generated by 254 nm photolysis of precursor HOSOH (*C*_2_-**1**) in argon matrices at 3.5 K. Its IR as well as UV/vis spectroscopic identification was supported by deuteration and computations. The spectroscopic evidence for the existence of *C*_2*v*_-**3** suggests the relevance of the H_2_O + SO reaction in Earth’s atmosphere and in the interstellar medium. We also confirm a tale of caution, as noted by Schaefer et al.:^22^ inclusion of the ZPVE can be pivotal for describing delicate energy relationships. Fortunately, it appears that a lower level of theory for determining the geometry and ZPVE produces acceptable results when a coupled cluster energy correction is included for this system.

## Supplementary Material



## Notes

Experimental infrared spectra, calculated structures, and vibrational frequencies (PDF)
