# Supplementary material for: The Sulfur Monoxide–Water Complex
Source: J Am Chem Soc. 2026 Jul 8;148(28):29635–40. doi: 10.1021/jacs.6c08436 (PMC13397562; doi:10.1021/jacs.6c08436)
Supplement: Supplementary file 1 [file ja6c08436_si_001.pdf]

Supporting Information

The Sulfur Monoxide-Water Complex

Guohai Deng,[1] Stephen M. Goodlett,[1] Caio M. Porto,[1] Artur Mardyukov,[1] and Peter R. Schreiner[1]*

[1]Institute of Organic Chemistry, Justus Liebig University, Heinrich-Buff-Ring 17, 35392 Giessen, Germany.

Table of Contents

Table of Contents S1

Additional experimental details, materials, and methods ....SError! Bookmark not defined.

Computational Methods S2

Figures S1-S3 .... S4

Tables S1-S4 .... S6

References S8

Optimized structures S13

Additional experimental details, materials, and methods

For the matrix isolation studies, we used an RDK 408D2 closed-cycle refrigerator cold head and an F-70 compressor system equipped with an inner polished CsI window for IR measurements. IR spectra were recorded between 7000 and 350 cm^{-1} with a resolution of 0.7 cm^{-1} with a Bruker Vertex 70 FTIR spectrometer and UV/vis spectra were recorded with a

JASCO V-670 spectrophotometer equipped with an inner sapphire window. A high-pressure-mercury lamp equipped with a monochromator (LOT Quantum Design) or a low-pressure-mercury lamp (Gräntzel) fitted with a Vycor filter were used for irradiation of the matrix during photochemical experiments.

For the high-vacuum flash pyrolysis experiment, we used a home-built, water-cooled oven, which was directly connected to the vacuum shroud of the cryostat. The pyrolysis zone was equipped with a heatable 90 mm long quartz tube (inner diameter 7 mm), controlled by a Ni/CrNi thermocouple. The travel distance of the sample from the pyrolysis zone to the matrix was ~45 mm. Ar was stored in a 2 L gas balloon, which was evacuated and filled three times before every experiment. *Tert*-butylsulfinic acid (**2**) was evaporated from a Schlenk tube at 26 °C (water) and reduced pressure (~3 × 10^6 mbar) and co-deposited with a high excess of argon on both sides of the matrix window in the dark (preventing unwanted photochemistry) at a rate of ~1 mbar min^{-1}, based on the pressure inside the Ar balloon. Pyrolyses were carried out at 600 °C. *t*-BuS(O)OH was synthesis by the reaction of *t*-BuS(O)Cl with excessive H_2O. The produced HCl and excessive H_2O were removed from the mixture under reduced pressure. ^{1}H-NMR (400 MHz, $CDCl_3$): δ/ppm = 11.28, 1.06. ^{13}C-NMR (400 MHz, $CDCl_3$): δ/ppm = 56.64, 22.30. *t*-BuS(O)OD was prepared using a similar method with D_2O.

Computational Methods

The noncovalent interaction (NCI) analysis was performed with the NCIPLOT program, using wavefunction files (.wfn).[1-2] They were obtained from the optimized structure of the adduct and computed separately for each molecule at the B3LYP(D3BJ)/def2-TZVP level.[3-5] The color-mapped RDG graph was made using the GNUPLOT software and the gradient isosurface (0.5 a.u.) was rendered by the VMD 1.9.3 program.[6]

For the focal point approach (FPA) energies, C_2-**1**, C_s-**1**, C_{2v}-**3**, and C_s-**4** were optimized using frozen core CCSD(T)[7-11]/aug-cc-pV(T+d)Z[12-15] in ORCA.[16-18] SCF energies and densities were converged to 10^{-10} a.u., and the optimizations converged to 10^{-7} for the energy and 10^{-5} a.u. for the RMS gradient and displacement. Unrestricted Hartree−Fock was used for all open shell species, as well as unrestricted coupled cluster theory. Frequencies were computed with the same method and basis as the optimization, indicating stationary points for all structures and yielding the zero-point vibrational energies (δ_{ZPVE}). Second order vibrational perturbation theory (vide infra) was used to obtain anharmonic corrections (δ_{AZPVE}) to the harmonic zero-

point vibrational energy. The focal point approach[19-21] was used to obtain relative energies of the species with respect to SO ($^3\Sigma^-$) and H_2O, where all energies were computed using the CCSD(T)/aug-cc-pV(T+d)Z structure. The shorthand AXZ refers to the basis set aug-cc-pV(X+d)Z, where X=D, T, Q, 5. CCSDT[22-24] and CCSDT(Q)[25-27] energies were computed with an ADZ basis using MRCC.[28-29] Basis set extrapolations were performed using a three point scheme[30-31] for SCF energies and a two point scheme[32] for correlation energies. Core-valence corrections (δ_{Core}) were made by calculating the difference between all electron and frozen core CCSD(T)/aug-cc-pCVTZ[12-14, 33-34] energies. Relativistic corrections ($\delta_{Rel.}$) were calculated as a difference between the all-electron Douglass–Kroll–Hess[35-36] Hamiltonian with CCSD(T)/aug-cc-pCVTZ-DK[12-14, 33-34, 37] and all-electron CCSD(T)/aug-cc-pCVTZ energies. Diagonal Born–Oppenheimer corrections[38] (δ_{DBOC}) were computed with Hartree–Fock/ATZ. Basis sets were obtained from the Basis Set Exchange.[39-41] Anharmonic frequencies computed with second order vibrational perturbation theory using finite difference of all electron MP2/ATZ analytic Hessians in Gaussian.[42] Differences between the MP2 harmonic and anharmonic frequencies were used to approximate the frozen core CCSD(T)/ATZ anharmonic frequencies. Table S3 shows the first ten excited states for each of the investigated species computed with EOM-CCSD[43-44]/aug-cc-pV(T+d)Z//CCSD(T)/aug-cc-pV(T+d)Z with Gaussian.

DFT computations were performed with ORCA, using the predefined "DefGrid3" integration grid. SCF energies and optimizations were converged to the same thresholds previously described for the FPA structures. For Figure 5 in the main text, the B3LYP,[3-4] B2PLYP,[45] ωB97X,[46-47] ωB97M,[48] and M06-2X[49] functionals were used with and without the D3,[50-51] D4,[52-54] and VV10[55] dispersion corrections and with the aug-cc-pV(T+d)Z basis set. The potential energy surface in Figure 6 was computed with CCSD(T)/aug-cc-pV(T+d)Z//B3LYP-D4/aug-cc-pV(T+d)Z with ZPVE energies from the geometry method.

NEVPT2 computations for excited state energies performed with ORCA using the strongly contracted variation with state averaging. For these, six electrons in six orbitals were considered. Natural bonding orbital (NBO) and natural energy decomposition analysis (NEDA) were done with NBO7. NEDA indicates Cs-4 has a higher stabilization from charge transfer interactions, in line with the results from LED.

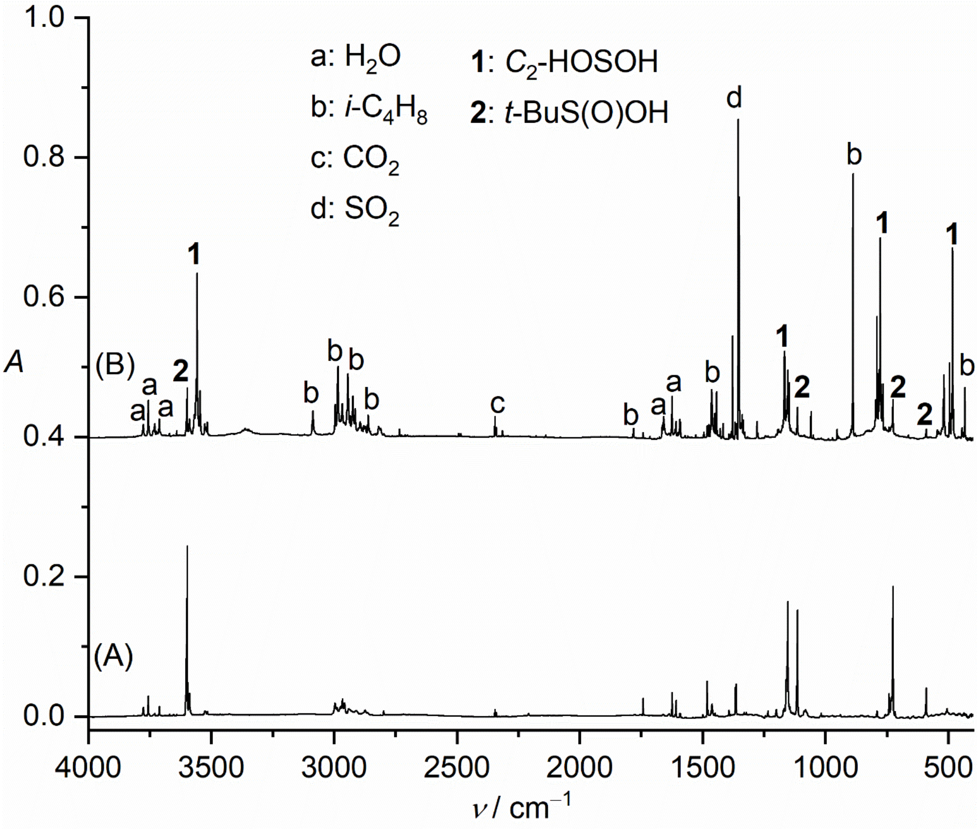

Figure S1. (A) IR spectrum for **2** in Ar-matrix at 3.5 K. (B) IR spectrum showing the product of pyrolysis of **2** with subsequent trapping in an argon matrix at 3.5 K.

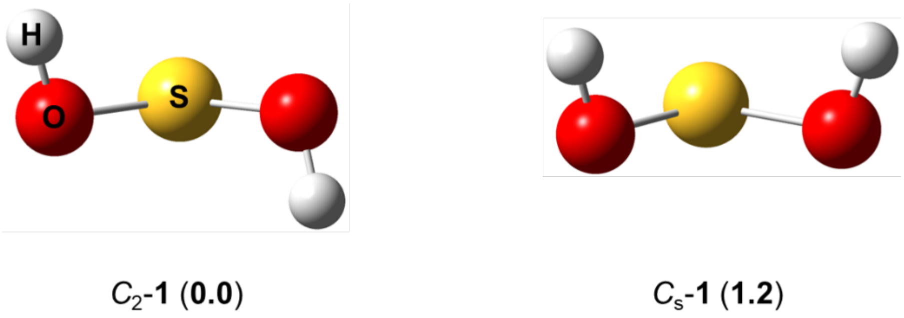

Figure S2. CCSD(T)/aug-cc-pV(T+d)Z computed structures and relative energies D_0 (kcal mol^{-1}) for the two HOSOH isomers.

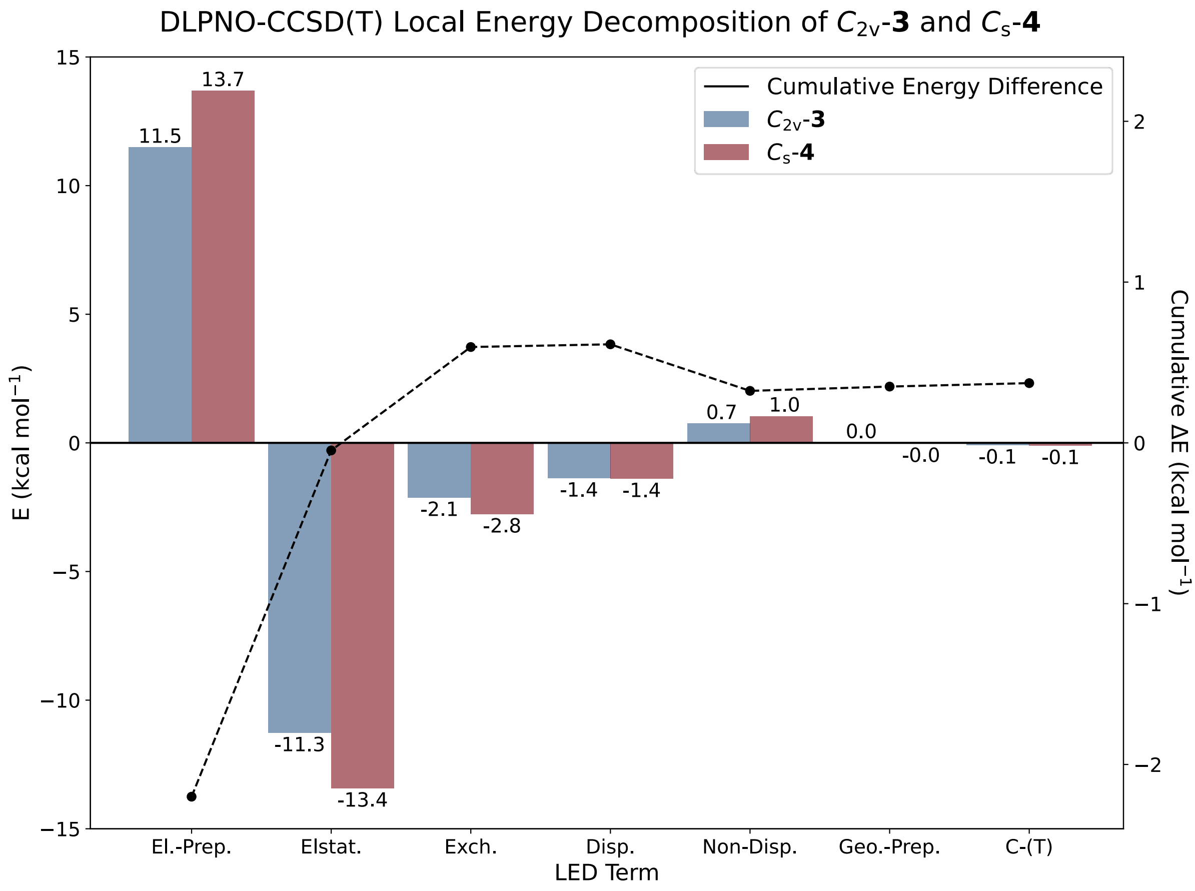

Figure S3. Local energy decomposition (DLPNO-CCSD(T)/aug-cc-pV(T+d)Z) analysis of the triplet minima. Contributions to C_{2v}-**3** (blue) and C_s-**4** (red) are plotted in bars and the cumulative interaction energy (added from left to right) is included (black). Abbreviated terms in order are the electronic preparation energy, the electrostatic interaction energy, the exchange interaction energy (this is not the Pauli exchange interaction, but the energy from electrons of same spin interacting), the dispersion energy, the non-dispersion energy in the DLPNO-CCSD correlation energy, the geometric preparation energy, and the perturbative triples correlation energy difference.

Table S1. B3LYP-D3(BJ)/def2-TZVP computed vibrational data of the C_2-**1**, C_s-**1**, C_{2v}-**3**, and C_s-**4**.

C_2-**1**	C_s-**1**	C_{2v}-**3**	C_s-**4**
3730.4 (13)	3734.9 (31)	3890.7 (60)	3874.6 (107)
3726.7 (107)	3732.1 (77)	3786.4 (13)	3759.7 (74)
1178.5 (50)	1174.8 (32)	1617.5 (79)	1622.2 (77)
1178.3 (20)	1161.3 (61)	1146.8 (30)	1159.1 (26)
789.4 (56)	787.4 (54)	177.1 (58)	314.4 (140)
774.1 (190)	774.6 (200)	109.9 (112)	235.6 (126)
524.8 (101)	548.1 (119)	103.3 (127)	142.3 (6)
512.5 (143)	458.3 (59)	62.9 (<1)	23.9 (23)
346.2 (<1)	353.0 (<1)	62.8 (48)	14.8 (128)

Table 2. Computed IR frequencies of **4** and ^{2}H-**4** (above 300 cm^{-1}) at the using CCSD(T)/aug-cc-pV(T+d)Z corrected MP2/aug-cc-pV(T+d)Z second order vibrational perturbation theory (VPT2) [Band Origins (cm^{-1}) (anharmonic) and Computed Intensities (km mol^{-1}) in Parentheses]. Note the first OH stretch corresponds to the antisymmetric stretch of water, likewise the second OH stretch corresponds to the symmetric stretch.

Mode	C_s-**4**	C_s-^{2}H-**4**	Assignment
B_2	3725.3 (108)	3695.2 (35)	OH str.
A_1	3628.9 (51)	2687.1 (70)	O(H/D) str.
A_1	1599.6 (55)	1400.3 (44)	HOH bend.
A_1	1159.9 (61)	1159.6 (62)	SO str.

Table S3. EOM-CCSD/aug-cc-pV(T+d)Z calculated vertical transitions for C_2-**1**, C_s-**1**, **3** and **4**.

C_2-**1**		C_s-**1**		C_{2v}-**3**		C_s-**4**	
λ/nm	oscillator strength	λ/nm	oscillator strength	λ/nm	oscillator strength	λ/nm	oscillator strength
264	0.0001	262	0.0006	256	0.0000	253	0.0000
234	0.0064	241	0.0070	256	0.0000	253	0.0000

193	0.0159	197	0.0162	248	0.0000	247	0.0002
182	0.0131	175	0.0007	237	0.0018	246	0.0001
159	0.0008	164	0.0348	237	0.0017	245	0.0000
158	0.0400	161	0.0013	212	0.0015	206	0.0013
157	0.0233	157	0.0814	211	0.0015	205	0.0025
156	0.0467	157	0.0241	193	0.1147	193	0.1093

Table S4. Focal point approach tables for C_2-**1** C_s-**1**, C_{2v}-**3**, and C_s-**4**. Energies are with respect to separated SO ($^3\Sigma^-$) and H_2O. Final values given as $\Delta H_{0K} = \Delta E_{FCI/CBS} + \delta_{ZPVE} + \delta_{AZPVE} + \delta_{Core} + \delta_{DBOC} + \delta_{Rel.}$ as described in the computational methods section.

	HF	δMP2	δCCSD	δCCSD(T)	δCCSDT	δCCSDT(Q)	[FCI]
C_2-**1**							
ADZ	4.96	-12.75	0.59	-0.99	0.20	0.11	[-7.89]
ATZ	4.30	-16.64	1.48	-1.00	[0.20]	[0.11]	[-11.55]
AQZ	4.25	-17.95	1.65	-1.00	[0.20]	[0.11]	[-12.74]
A5Z	4.31	-18.47	1.84	[-1.08]	[0.20]	[0.11]	[-13.10]
[CBS]	[4.35]	[-19.02]	[2.05]	[-1.17]	[0.20]	[0.11]	[-13.48]
	$\Delta H_{0K} = -13.48 + 3.41 - 0.08 - 0.27 + 0.00 + 0.18 = \mathbf{-10.24}$						
C_s-**1**							
ADZ	6.46	-13.01	0.63	-1.04	0.19	0.11	[-6.67]
ATZ	5.75	-16.88	1.52	-1.05	[0.19]	[0.11]	[-10.36]
AQZ	5.69	-18.19	1.69	-1.05	[0.19]	[0.11]	[-11.55]
A5Z	5.74	-18.70	1.89	[-1.13]	[0.19]	[0.11]	[-11.91]
[CBS]	[5.78]	[-19.24]	[2.09]	[-1.22]	[0.19]	[0.11]	[-12.29]
	$\Delta H_{0K} = -12.29 + 3.38 - 0.09 - 0.26 + 0.00 + 0.18 = \mathbf{-9.07}$						
C_{2v}-**3**							
ADZ	-1.84	-1.17	0.22	-0.16	0.03	0.00	[-2.93]
ATZ	-1.45	-1.39	0.28	-0.19	[0.03]	[0.00]	[-2.75]
AQZ	-1.39	-1.42	0.30	-0.19	[0.03]	[0.00]	[-2.71]
A5Z	-1.37	-1.38	0.30	[-0.23]	[0.03]	[0.00]	[-2.69]
[CBS]	[-1.36]	[-1.35]	[0.30]	[-0.27]	[0.03]	[0.00]	[-2.66]
	$\Delta H_{0K} = -2.66 + 0.62 - 0.03 - 0.07 + 0.0 - 0.03 = \mathbf{-2.17}$						
C_s-**4**							
ADZ	-1.69	-1.79	0.47	-0.26	0.02	0.02	[-3.23]
ATZ	-1.53	-1.86	0.56	-0.25	[0.02]	[0.02]	[-3.05]
AQZ	-1.52	-1.76	0.60	-0.24	[0.02]	[0.02]	[-2.88]
A5Z	-1.49	-1.71	0.60	[-0.24]	[0.02]	[0.02]	[-2.79]
[CBS]	[-1.47]	[-1.65]	[0.60]	[-0.24]	[0.02]	[0.02]	[-2.72]
	$\Delta H_{0K} = -2.72 + 1.11 - 0.14 - 0.06 - 0.01 + 0.0 = \mathbf{-1.82}$						

References

(1) Johnson, E. R.; Keinan, S.; Mori-Sánchez, P.; Contreras-García, J.; Cohen, A. J.; Yang, W. T. Revealing Noncovalent Interactions. *J. Am. Chem. Soc.* **2010,** *132*, 6498−6506.

(2) Contreras-García, J.; Johnson, E. R.; Keinan, S.; Chaudret, R.; Piquemal, J. P.; Beratan, D. N.; Yang, W. T. NCIPLOT: A Program for Plotting Noncovalent Interaction Regions. *J. Chem. Theory Comput.* **2011,** *7*, 625−632.

(3) Becke, A. D. Density-Functional Thermochemistry. III. The Role of Exact Exchange. *J. Chem. Phys.* **1993**, *98*, 5648–5652.

(4) Stephens, P. J.; Devlin, F. J.; Chabalowski, C. F.; Frisch, M. J. Ab initio Calculation of Vibrational Absorption and Circular Dichroism Spectra using Density Functional Force Fields. *J. Phys. Chem.* **1994**, *98*, 11623–11627.

(5) Weigend, F.; Ahlrichs, R. Balanced Basis Sets of Split Valence, Triple Zeta Valence and Quadruple Zeta Valence Quality for H to Rn: Design and Assessment of Accuracy. *Phys. Chem. Chem. Phys.* **2005**, *7*, 3297–3305.

(6) Humphrey, W.; Dalke, A.; Schulten, K. VMD: Visual Molecular Dynamics. *J. Molec. Graphics* **1996**, *14*.1, 33−38.

(7) Purvis, G. D.; Bartlett, R. J. A Full Coupled-Cluster Singles and Doubles Model: The Inclusion of Disconnected Triples. *J. Chem. Phys.* **1982**, *76*, 1910–1918.

(8) Raghavachari, K.; Trucks, G. W.; Pople, J. A.; Head-Gordon, M. A Fifth-Order Perturbation Comparison of Electron Correlation Theories. *Chem. Phys. Lett.* **1989**, *157*, 479–483.

(9) Bartlett, R. J.; Watts, J. D.; Kucharski, S. A.; Noga, J. Non-Iterative Fifth-Order Triple and Quadruple Excitation Energy Corrections in Correlated Methods. *Chem. Phys. Lett.* **1990**, *165*, 513–522.

(10) Knowles, P. J.; Hampel, C.; Werner, H. J. Coupled Cluster Theory for High Spin, Open Shell Reference Wave Functions. *J. Chem. Phys.* **1993**, *99*, 5219–5227.

(11) Stanton, J. F. Why CCSD(T) Works: A Different Perspective. *Chem. Phys. Lett.* **1997**, *281*, 130–134.

(12) Dunning, T. H. Gaussian Basis Sets for Use in Correlated Molecular Calculations. I. The Atoms Boron through Neon and Hydrogen. *J. Chem. Phys.* **1989**, *90*, 1007–1023.

(13) Kendall, R. A.; Dunning, T. H.; Harrison, R. J. Electron Affinities of the First-Row Atoms Revisited. Systematic Basis Sets and Wave Functions. *J. Chem. Phys.* **1992**, *96*, 6796–6806.

(14) Woon, D. E.; Dunning, T. H. Gaussian Basis Sets for Use in Correlated Molecular Calculations. III. The Atoms Aluminum through Argon. *J. Chem. Phys.* **1993**, *98*, 1358–1371.

(15) Dunning, T. H.; Peterson, K. A.; Wilson, A. K. Gaussian Basis Sets for Use in Correlated Molecular Calculations. X. The Atoms Aluminum through Argon Revisited. *J. Chem. Phys.* **2001**, *114*, 9244–9253.
(16) Neese, F.; Wennmohs, F.; Becker, U.; Riplinger, C. The ORCA Quantum Chemistry Program Package. *J. Chem. Phys.* **2020**, *152*, 224108.
(17) Neese, F. Software Update: The ORCA Program System—Version 5.0. *WIREs Comput. Mol. Sci.* **2022**, *12*, e1606.
(18) Neese, F. Software Update: The ORCA Program System—Version 6.0. *WIREs Comput. Mol. Sci.* **2025**, *15*, e70019.
(19) East, A. L. L.; Allen, W. D. The Heat of Formation of NCO. *J. Chem. Phys.* **1993**, *99*, 4638–4650.
(20) Császár, A. G.; Allen, W. D.; Schaefer, H. F., III. In Pursuit of the ab initio Limit for Conformational Energy Prototypes. *J. Chem. Phys.* **1998**, *108*, 9751–9764.
(21) Schuurman, M. S.; Muir, S. R.; Allen, W. D.; Schaefer, H. F., III. Toward Subchemical Accuracy in Computational Thermochemistry: Focal Point Analysis of the Heat of Formation of NCO and [H, N, C, O] Isomers. *J. Chem. Phys.* **2004**, *120*, 11586–11599.
(22) Noga, J.; Bartlett, R. J. The Full CCSDT Model for Molecular Electronic Structure. *J. Chem. Phys.* **1987**, *86*, 7041–7050.
(23) Scuseria, G. E.; Schaefer, H. F. A New Implementation of the Full CCSDT Model for Molecular Electronic Structure. *Chem. Phys. Lett.* **1988**, *152*, 382–386.
(24) Kállay, M.; Surján, P. R. Higher Excitations in Coupled-Cluster Theory. *J. Chem. Phys.* **2001**, *115*, 2945–2954.
(25) Bomble, Y. J.; Stanton, J. F.; Kállay, M.; Gauss, J. Coupled-Cluster Methods Including Noniterative Corrections for Quadruple Excitations. *J. Chem. Phys.* **2005**, *123*, 054101.
(26) Kállay, M.; Gauss, J. Approximate Treatment of Higher Excitations in Coupled-Cluster Theory. *J. Chem. Phys.* **2005**, *123*, 214105.
(27) Kállay, M.; Gauss, J. Approximate Treatment of Higher Excitations in Coupled-Cluster Theory. II. Extension to General Single-Determinant Reference Functions and Improved Approaches for the Canonical Hartree-Fock Case. *J. Chem. Phys.* **2008**, *129*, 144101.
(28) MRCC, A Quantum Chemical Program Suite written by M. Kállay, P. R. N., D. Mester, Z. Rolik, G. Samu, J. Csontos, J. Csóka, P. B. Szabó, L. Gyevi-Nagy, B. Hégely, I. Ladjánszki, L. Szegedy, B. Ladóczki, K. Petrov, M. Farkas, P. D. Mezei, and A. Ganyecz. See www.mrcc.hu.

(29) Mester, D.; Nagy, P. R.; Csóka, J.; Gyevi-Nagy, L.; Szabó, P. B.; Horváth, R. A.; Petrov, K.; Hégely, B.; Ladóczki, B.; Samu, G.; Lőrincz, B. D.; Kállay, M. Overview of Developments in the MRCC Program System. *J. Phys. Chem. A* **2025**, *129*, 2086–2107.

(30) Feller, D. The Use of Systematic Sequences of Wave Functions for Estimating the Complete Basis Set, Full Configuration Interaction Limit in Water. *J. Chem. Phys.* **1993**, *98*, 7059– 7071.

(31) Feller, D.; Peterson, K. A.; Crawford, T. D. Sources of Error in Electronic Structure Calculations on Small Chemical Systems. *J. Chem. Phys.* **2006**, *124*, 054107.

(32) Helgaker, T.; Klopper, W.; Koch, H.; Noga, J. Basis-set Convergence of Correlated Calculations on Water. *J. Chem. Phys.* **1997**, *106*, 9639–9646.

(33) Woon, D. E.; Dunning, T. H. Gaussian Basis Sets for Use in Correlated Molecular Calculations. V. Core-Valence Basis Sets for Boron through Neon. *J. Chem. Phys.* **1995**, *103*, 4572–4585.

(34) Peterson, K. A.; Dunning, T. H. Accurate Correlation Consistent Basis Sets for Molecular Core-Valence Correlation Effects: The Second Row Atoms Al-Ar, and the First Row Atoms B-Ne Revisited. *J. Chem. Phys.* **2002**, *117*, 10548–10560.

(35) Douglas, M.; Kroll, N. M. Quantum Electrodynamical Corrections to the Fine Structure of Helium. *Ann. Phys.* **1974**, *82*, 89–155.

(36) Hess, B. A. Relativistic Electronic-structure Calculations Employing a Two-Component No-Pair Formalism with External-Field Projection Operators. *Phys. Rev. A* **1986**, *33*, 3742–3748.

(37) de Jong, W. A.; Harrison, R. J.; Dixon, D. A. Parallel Douglas–Kroll Energy and Gradients in NWChem: Estimating Scalar Relativistic Effects using Douglas–Kroll Contracted Basis Sets. *J. Chem. Phys.* **2001**, *114*, 48–53.

(38) Handy, N. C.; Yamaguchi, Y.; Schaefer, H. F. The Diagonal Correction to the Born–Oppenheimer Approximation: Its Effect on the Singlet–Triplet Splitting of CH_2 and Other Molecular Effects. *J. Chem. Phys.* **1986**, *84*, 4481–4484.

(39) Feller, D. The Role of Databases in Support of Computational Chemistry Calculations. *J. Comput. Chem.* **1996**, *17*, 1571–1586.

(40) Schuchardt, K. L.; Didier, B. T.; Elsethagen, T.; Sun, L. S.; Gurumoorthi, V.; Chase, J.; Li, J.; Windus, T. L. Basis Set Exchange: A Community Database for Computational Sciences. *J. Chem. Inf. Model.* **2007**, *47*, 1045–1052.

(41) Pritchard, B. P.; Altarawy, D.; Didier, B.; Gibson, T. D.; Windus, T. L. A New Basis Set Exchange: An Open, Up-to-Date Resource for the Molecular Sciences Community. *J. Chem. Inf. Model.* **2019**, *59*, 4814–4820.

(42) Frisch, M. J.; Trucks, G. W.; Schlegel, H. B.; Scuseria, G. E.; Robb, M. A.; Cheeseman, J. R.; Scalmani, G.; Barone, V.; Petersson, G. A.; Nakatsuji, H.; Li, X.; Caricato, M.; Marenich, A. V.; Bloino, J.; Janesko, B. G.; Gomperts, R.; Mennucci, B.; Hratchian, H. P.; Ortiz, J. V.; Izmaylov, A. F.; Sonnenberg, J. L.; Williams-Young, D.; Ding, F.; Lipparini, F.; Egidi, F.; Goings, J.; Peng, B.; Petrone, A.; Henderson, T.; Ranasinghe, D.; Zakrzewski, V. G.; Gao, J.; Rega, N.; Zheng, G.; Liang, W.; Hada, M.; Ehara, M.; Toyota, K.; Fukuda, R.; Hasegawa, J.; Ishida, M.; Nakajima, T.; Honda, S22 Y.; Kitao, O.; Nakai, H.; Vreven, T.; Throssell, K.; Montgomery, J. A., Jr.; Peralta, J. E.; Ogliaro, F.; Bearpark, M. J.; Heyd, J. J.; Brothers, E. N.; Kudin, K. N.; Staroverov, V. N.; Keith, T. A.; Kobayashi, R.; Normand, J.; Raghavachari, K.; Rendell, A. P.; Burant, J. C.; Iyengar, S. S.; Tomasi, J.; Cossi, M.; Millam, J. M.; Klene, M.; Adamo, C.; Cammi, R.; Ochterski, J. W.; Martin, R. L.; Morokuma, K.; Farkas, O.; Foresman, J. B.; Fox, D. J. *Gaussian 16, C1*; **2016**, Gaussian Inc.: Wallingford, CT.

(43) Koch, H.; Jorgensen, P. Coupled Cluster Response Functions. *J. Chem. Phys.* **1990**, *93*, 3333–3344.

(44) Stanton, J. F.; Bartlett, R. J. The Equation of Motion Coupled-Cluster Method. A Systematic Biorthogonal Approach to Molecular Excitation Energies, Transition Probabilities, and Excited State Properties. *J. Chem. Phys.* **1993**, *98*, 7029–7039.

(45) Grimme, S. Semiempirical Hybrid Density Functional with Perturbative Second-Order Correlation. *J. Chem. Phys.* **2006**, *124*, 034108.

(46) Chai, J.-D.; Head-Gordon, M. Systematic Optimization of Long-Range Corrected Hybrid Density Functionals. *J. Chem. Phys.* **2008**, *128*, 084106.

(47) Najibi, A.; Goerigk, L. DFT-D4 Counterparts of Leading Meta-Generalized-Gradient Approximation and Hybrid Density Functionals for Energetics and Geometries. *J. Comput. Chem.* **2020**, *41*, 2562–2572.

(48) Mardirossian, N.; Head-Gordon, M. ωB97M-V: A Combinatorially Optimized, Range-Separated Hybrid, Meta-GGA Density Functional with VV10 Nonlocal Correlation. *J. Chem. Phys.* **2016**, *144*, 214110.

(49) Zhao, Y.; Truhlar, D. The M06 Suite of Density Functionals for Main Group Thermochemistry, Thermochemical Kinetics, Noncovalent Interactions, Excited States, and

Transition Elements: Two New Functionals and Systematic Testing of Four M06-Class Functionals and 12 Other Functionals. *Theor. Chem. Acc.* **2008**, *120*, 215–241.
(50) Grimme, S.; Antony, J.; Ehrlich, S.; Krieg, H. A Consistent and Accurate ab initio Parametrization of Density Functional Dispersion Correction (DFT-D) for the 94 Elements H-Pu. *J. Chem. Phys.* **2010**, *132*, 154104.
(51) Grimme, S.; Ehrlich, S.; Goerigk, L. Effect of the Damping Function in Dispersion Corrected Density Functional Theory. *J. Comput. Chem.* **2011**, *32*, 1456–1465.
(52) Caldeweyher, E.; Bannwarth, C.; Grimme, S. Extension of the D3 Dispersion Coefficient Model. *J. Chem. Phys.* **2017**, *147*, 034112.
(53) Caldeweyher, E.; Ehlert, S.; Hansen, A.; Neugebauer, H.; Spicher, S.; Bannwarth, C.; Grimme, S. A Generally Applicable Atomic-Charge Dependent London Dispersion Correction. *J. Chem. Phys.* **2019**, *150*, 154122.
(54) Caldeweyher, E.; Mewes, J.-M.; Ehlert, S.; Grimme, S. Extension and Evaluation of the D4 London-Dispersion Model for Periodic Systems. *Phys. Chem. Chem. Phys.* **2020**, *22*, 8499–8512.
(55) Vydrov, O. A.; Van Voorhis, T. Nonlocal van Der Waals Density Functional: The Simpler the Better. *J. Chem. Phys.* **2010**, *133*, 244103.

Computed atomic coordinates (in Å) and spin quantum number (S) of species for optimized structures at B3LYP-D4/aug-cc-pV(T+d)Z. Additionally reported are the spin expectation value (S^2) and T_1 diagnostic for CCSD(T)/aug-cc-pV(T+d)Z.

HOSOH (C_2-**1**) S=0, S^2=0.0, T_1=0.016

S	0.000014	0.000001	0.562498
O	-0.079619	-1.295642	-0.458532
O	0.079614	1.295667	-0.458507
H	0.816011	-1.544244	-0.723532
H	-0.816020	1.544218	-0.723548

HOSOH (C_2-**1**) S=1, S^2=2.044, T_1=0.026

S	-0.102406	0.201365	0.944697
O	-0.499289	1.445352	-0.220817
O	0.443766	-1.369100	1.491146
H	-0.351718	1.164235	-1.134302
H	0.692530	-1.954102	0.762345

HOSOH (C_s-**1**) S=0, S^2=0.0, T_1=0.0159

S	0.046491	0.618374	0.274240
O	-1.506531	0.220466	-0.123292
O	0.955220	-0.556464	-0.448412
H	-1.876647	-0.355719	0.557891
H	1.083238	-1.289868	0.166987

OS•••OH_2 (C_{2v}-**3**) S=1, S^2=2.058, T_1=0.0172

S	-0.000001	0.000000	-0.480631
O	-0.000003	0.000000	2.528717
O	0.000003	-0.000000	-1.972787
H	-0.765231	-0.000000	3.111907
H	0.765233	-0.000000	3.111898

OS•••OH_2 (C_s-**3**) S=0, S^2=0.0, T_1=0.0173

S	0.658685	-0.038665	-0.000000
O	0.079274	2.239938	0.000000
O	-0.643321	-0.773975	0.000000
H	-0.492985	2.323808	-0.772909
H	-0.492985	2.323808	0.772909

SO•••H_2O (C_s-**4**) S=1, S^2=2.055, T_1=0.0175

S	-2.724444	0.000000	-3.484991
O	-3.233358	-0.000000	-2.084741
O	-1.065823	-0.000000	0.009642
H	-1.909302	-0.000000	-0.458791
H	-1.293427	0.000000	0.943713

HOS(O)H S=0, S^2=0.0, T_1=0.0185

S	-0.147970	0.402645	-0.138814

O	1.323664	-0.320681	0.149245
O	-1.241536	-0.543029	0.088933
H	1.630483	-0.719378	-0.676261
H	0.017338	1.087573	1.041458

OS(H) •••OH, S=1, S^2=2.090, T_1=0.0351

S	-0.152578	0.059676	-0.000173
O	-1.657735	-0.163254	-0.000002
O	1.715020	-0.011889	-0.000361
H	-0.004322	1.399736	-0.000241
H	2.002449	-0.936533	-0.000325

HOSO S=1/2, S^2=0.785, T_1=0.0216

S	-0.199860	0.067922	0.480241
O	0.024019	-1.395093	-0.238139
O	0.249008	1.133352	-0.433607
H	0.739951	-1.332523	-0.890166

HOS S=1/2, S^2=0.768, T_1=0.0200

S	0.021880	0.004445	0.474532
O	0.052231	1.401469	-0.384952
H	0.307248	1.206754	-1.297318

OH S=1/2, S^2=0.757, T_1=0.0103

O	-0.037806	0.000000	0.000000
H	0.937806	0.000000	0.000000

H_2O S=0, S^2=0.0, T_1=0.0101

O	-1.015825	-0.000000	-2.261793
H	-1.015825	0.763690	-1.676539
H	-1.015825	-0.763690	-1.676539

TS1 S=0, S^2=0.0, T_1=0.0193

S	-0.400327	-0.597776	0.010787
O	1.324214	0.133587	-0.105923
O	-0.807612	0.929615	0.010584
H	1.768779	0.048969	0.752755
H	0.554186	1.008791	-0.066315

TS2 S=0, S^2=0.0, T_1=0.0232

S	-0.058639	0.485612	-0.049190
O	1.325198	-0.400995	0.120800
O	-1.318889	-0.531921	-0.059929
H	1.712506	-0.550836	-0.753566
H	-0.812849	0.074549	1.079695

TS3 S=1, S^2=2.087, T_1=0.0325

S	-0.054192	-0.303962	0.000673
O	-1.680970	0.221900	0.055635
O	1.635776	0.109982	-0.047190
H	-0.610074	1.060255	0.026820

H 1.811666 1.060766 -0.038763

TS4 S=1, S^2=2.043, T_1=0.0249

S	0.436738	-0.194536	0.012028
O	-2.390367	0.042283	-0.095303
O	1.888068	0.199526	0.012706
H	-3.019551	0.172310	0.638111
H	-0.256047	0.988150	-0.099475

TS5 S=0, S^2=1.0, T1=0.0151

S	0.017621	-0.577330	0.009756
O	1.265306	0.466919	-0.110356
O	-1.229452	0.565749	-0.009795
H	1.493202	0.795600	0.769998
H	-2.066552	0.084007	0.036172

CCSD(T)/aug-cc-pV(T+d)Z coordinates in Ångstrom.

HOSOH (C_2-**1**) S=0, S^2=0.0, T_1=0.0158

S	-0.000011	-0.000001	0.564039
O	-0.086965	-1.287311	-0.462860
O	0.086969	1.287287	-0.462884
H	-0.813119	1.526341	-0.719951
H	0.813126	-1.526316	-0.719966

HOSOH (C_s-**1**) S=0, S^2=0.0, T_1=0.0158

S	0.047333	0.619545	0.277879
O	-1.499947	0.217824	-0.127964
O	0.947399	-0.554559	-0.451155
H	-1.855084	-0.362671	0.556963
H	1.062070	-1.283351	0.171691

OS•••OH_2 (C_{2v}-**3**) S=1, S^2=2.058, T_1=0.0173

S	-0.000001	0.000000	-0.481738
O	-0.000003	0.000000	2.526227
O	0.000003	-0.000000	-1.976730
H	-0.760068	-0.000000	3.115677
H	0.760070	-0.000000	3.115668

SO•••H_2O (C_s-**4**) S=1, S^2=2.056, T_1=0.0176

S	-2.649670	0.000000	-3.429075
O	-3.283752	-0.000000	-2.077050
O	-1.079449	0.000000	-0.031814
H	-1.948653	-0.000000	-0.448532
H	-1.264830	-0.000000	0.911303

H_2O S=0, S^2=0.0, T_1=0.0101

O	0.111858	0.000000	0.000000
H	0.702673	0.000000	0.758669
H	0.702673	0.000000	-0.758669

SO S=1, S^2=2.060, T_1=0.0208

S	-0.005691	0.000000	0.000000
O	1.486691	0.000000	0.000000
